# ATP-Binding Cassette Transporter Regulates N,N′-diacetylchitobiose Transportation and Chitinase Production in *Trichoderma asperellum* T4

**DOI:** 10.3390/ijms20102412

**Published:** 2019-05-15

**Authors:** He Liu, Ming Cheng, Shanshan Zhao, Congyu Lin, Jinzhu Song, Qian Yang

**Affiliations:** School of Life Science and Technology 150080, Harbin Institute of Technology, Harbin 150000, China; HIT.microbiolab207@hotmail.com (H.L.); glxmdj@163.com (M.C.); zhaoshanshan5612@163.com (S.Z.); lincongyu@hit.edu.cn (C.L.); sjz@hit.edu.cn (J.S.)

**Keywords:** ABC transporter, chitobiose, chitinase, ECH42, *Trichoderma asperellum*

## Abstract

ATP-binding cassette (ABC) transporters are a superfamily of proteins that transport nutrient substances and secondary metabolites through cell membranes. They also act as an uptake system for N,N′-diacetylchitobiose (GlcNAc)_2_ in *Streptomyces coelicolor*. (GlcNAc)_2_ is an important inducer of chitinase. However, whether the ABC transporter in *Trichoderma* spp. is also responsible for (GlcNAc)_2_ uptake and chitinase induction has not yet been confirmed. In this study, we applied RNA interference and overexpression technologies to alter the expression level of the ABC-B transporter in order to detect changes in its transportation ability and the expression level of inducible endo-chitinase ECH42—an important biocontrol enzyme in *Trichoderma asperellum*. The results revealed that, after interference with the expression of the ABC-B transporter, *T. asperellum* T4 was only able to grow normally when glucose was the only carbon source. Compared with the wild-type, the efficiency of (GlcNAc)_2_ by the overexpression strain evidently increased, along with the activity level of ECH42. In conclusion, one of the functions of the ABC-B transporter in *T.* *asperellum* is the uptake and transport of (GlcNAc)_2_ into cells, and chitobiose is a strong inducer of ECH42 in *T. asperellum* T4.

## 1. Introduction

ATP-binding cassette (ABC) transporters are a super family of membrane proteins that use energy from ATP hydrolysis to transport substrates, such as small inorganic and organic molecules, metal ions, polypeptides, and proteins, through the cell wall [1,2,3]. Early stages of research have shown that the ABC transporter and its homologous proteins exist not only in bacteria but also in eukaryons and mammals. The ABC superfamily is the largest known protein family [4]. ABC proteins can be divided into two groups based on their functions: importers and exporters [5,6]. The first group can be further divided into importers I and II, whose main function is to transport nutrients from outer to inner regions, i.e., from the trans- to the cis-side [7,8]. Most exporters in bacteria and eukaryons, such as ABC-A/B/C/D, are associated with the exportation of toxic substances and phytohormones [9,10,11]. However, recent studies have shown that exporters such as ABC-B/D/G may also play the role of importers in eukaryons, which challenges the conclusion that the ABC importer is only found in bacteria. Thus, the full functionality of this family requires further research [12,13,14,15].

*Trichoderma asperellum* is a well-known biocontrol agent with significant activity against phytopathogens [16]. Hyperparasitism is one of the most important measures that protect plants against fungal diseases caused by *Pythium* and *Fusarium* spp. [17]. Chitin and chitinase play crucial roles during this process. Chitin is an important structural component of fungal cell walls that is required for the maintenance of structural integrity. Chitinase is the key enzyme involved in the physical penetration of phytopathogen cell walls, and plays an important role in cell mitosis [18,19,20,21]. To date, studies have mainly focused on (1) the use of the heterologous expression of the chitinase gene to improve plant resistance to pathogens and insects, or (2) the functions of chitinase and chitinase-like proteins in cancer [22,23,24]. Only a few papers have studied the regulation mechanisms of chitinase. Dimeric N-acetylglucosamine (GlcNAc)_2_ has been proven to strongly induce the expression of chitinase in several microorganisms, including bacteria and fungi such as *Streptomycetes* and the mushroom *Coprinopsis cinerea* [25,26]. However, it is unknown whether *T. asperellum* can also be induced or whether the ABC transporter is an agent that transports (GlcNAc)_2_ into the trichoderma cells. Endo-chitinase ECH42 (GenBank: GU457410.1) is an essential enzyme for biocontrol activities, which has no chitin-binding domain and can only be induced by insoluble chitins, such as (GlcNAc), (GlcNAc)_2_, (GlcNAc)_3_, or colloidal chitin [23,27]. In this study, RNA interference (RNAi) and overexpression technologies were applied on a genetic level to change the expression level of the ABC-B transporter, and the differences in the uptake of (GlcNAc)_2_, the expression level, and the activity of ECH42 were detected.

## 2. Results and Discussion

### 2.1. Characterization of the ABC-B Transport Genes in T. asperellum T4

**Figure 1:**
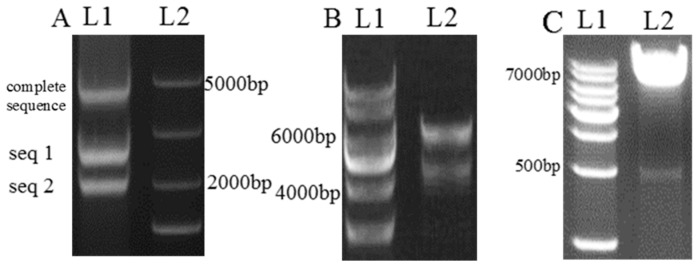
Construction of the *ABC* complete sequence, the pSilent-*ABC* plasmid, and the double enzyme digest result. (**A**) L1. Sequences 1 and 2 and the seamless connection results. L2. DNA marker. (**B**) L1. DNA marker. L2. Sequence of pBARGPE-1 and *ABC* after *XhoI* and *BamHI* enzyme digestion. (**C**) L1. DNA marker. L2. pSilent plasmid (7056 bp) and interference sequence (500 bp) after *HindIII* and *XhoI* digestion.

Using cDNA as a template, after 29 cycles of PCR, two sequences were acquired, ABCOVER-1 and ABCOVER-2, 1923 and 2451 bp long, respectively. These two sequences were 99% identical to the mRNA of the *ABC* sequence on NCBI, based on a BLAST compare. The ClonExpress II One Step Cloning Kit was chosen to connect sequences 1 and 2. As a result, a complete sequence of the *ABC* transporter cDNA was obtained (Figure 1A). ABC-I and ABC-II were connected to Psilent plasmid through the enzyme sites mentioned in (3.3.1). The complete overexpression sequence of *ABC* was first measured using a kit through the overlap section, which was then connected to the pBargpe plasmid through enzyme sites. The plasmids pSilent-*ABC* and pBargpe-Hygro-*ABC* were sequenced after double enzyme digestion (Figure 1B,C), and both sequences were found to be 99% identical to the NCBI template.

**Figure 2:**
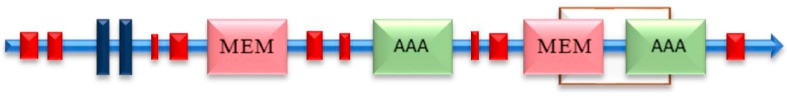
The structure of the ABC transporter on SMART. Red rectangles: regions of low compositional complexity. Blue rectangles: transmembrane helix regions. Pink rectangles: regions of the Pfam *ABC* membrane with transmembrane domains comprising six alpha helices. AAA regions: ATPases associated with various cellular activities.

AAA regions, which contain ATPases associated with a variety of cellular activities, such as membrane fusion, proteolysis, recombination, repair, transcription, and DNA replication, can be found in almost every living organism. However, in this vast family, only a fraction of this alignment was detected. The ABC membrane regions (MEM) shown in pink represent exporters with triple 2 transmembrane segment precursors. They can also be considered as conserved transmembrane domains that are usually encoded by independent polypeptides. They act as extracytoplasmic receptors (solute-binding proteins), with the primary function of translocating all kinds of substrates across the membrane. The ABC transporter formed in this study was of the ABC-B type based on its structure (Figure 2) [28,29]. The B subfamily is widely distributed in eukaryons owing to its various functions, such as chemical resistance, antigen synthesis, pheromone pump-out, and heavy metal tolerance [30,31]. A brown frame area of 500 bp was chosen as the interfered target, because it is the most crucial function zone of the protein. The designed primers are shown in Table 1.

### 2.2. Growth of Protoplasts and Expression Level of the ABC-B Transporter

**Figure 3:**
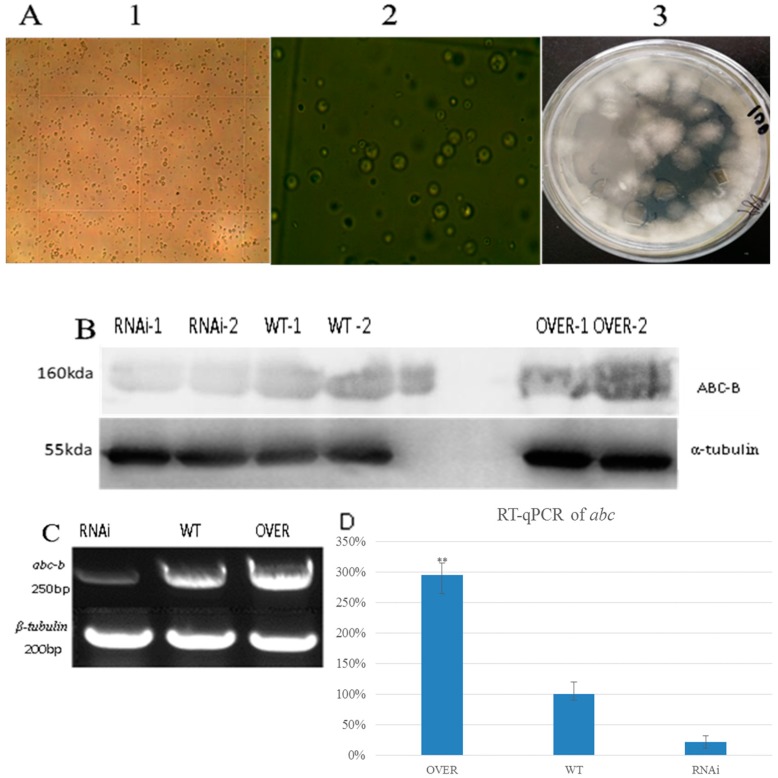
Protoplast state. Western blot of the expression level of ABC-B. Semi-quantitative and RT-qPCR results for the expression of ABC-B. (**A**1,2) Digestion of the cell wall and enrichment condition under 10× and 40× magnification. (3) Growth condition of protoplasts on agar plates. (**B**) Western blot result for ABC-B and the corresponding α-tubulin reference. RNAi-1,2: Expression level of protein ABC-B of the interfered strains selected from the plate. RNAi-1 was chosen as the most interfered strain. WT-1,2: Expression level of the wild-type (WT) groups. WT-2 was chosen as the standard. OVER-1,2: Protein level of ABC-B of the overexpressed strains. OVER-1 was chosen as the most overexpressed strain. (**C**) Semi-quantitative PCR results for ABC-B and the reference, β-tubulin. RNAi: Genetic expression level of ABC-B of the RNAi-1 strain. WT: Expression level of WT-2. OVER: Expression level of the overexpressed strain OVER-1. (**D**) Histogram of the RT-qPCR result with statistical analysis (*p* < 0.01).

Based on 10× and 40× magnification images, the quantity of *T. asperellum* protoplasts is >10^6^ as per the PEG-mediated plastid transformation (i.e., at least 20 protoplasts in one mid-chamber; Figure 3A). After two days of growth, the spores of the RNAi and overexpressed strains on the PDA agar plate were collected using a certain concentration of hygromycin B as a selective agent. They were cultured in MM for three days. Then, their RNA was extracted, and RT-qPCR was applied to identify the strain with the highest ABC-B interference or overexpression efficiency. The expression level of the ABC-B transporter in the RNAi strain was 22% of the WT expression level. Under the same conditions, the level of the ABC-B transporter in the overexpressed strain was found to be 95% greater than that of the WT (Figure 3C,D). The Western blot analysis revealed that the expression level of RNAi-1 strain was 21.7% of the level of WT-2, and the level of OVER-1 was 95% higher than level of WT-2. Though OVER-2 was 18.5 times more than WT-2 (Figure 3B), this result could not be repeated. At same time, the ABC level of OVER-1 was stable, so OVER-1 was chosen as the most overexpressed strain for subsequent experiments.

### 2.3. Growth Conditions Causing a Lack of ABC on Different Polysaccharides

**Figure 4:**
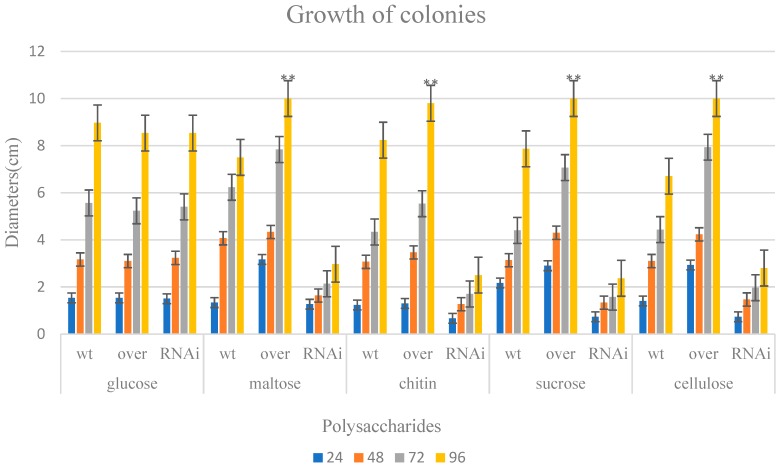
Growth of ABC-B interfered strains, overexpressed strains, and WT. The different colors indicate the hours of incubation. Except for glucose, the diameters of all other overexpressed strains were significantly increased compared with their respective interfered strains (*p* < 0.01).

Based on the known functions of ABC transporters, their ability to uptake polysaccharides, such as maltose, cellulose, chitin, and sucrose, could be influenced by genetic engineering. The growth conditions of the *ABC* RNAi and overexpressed strains were tested on MM agar plates using glucose, maltose, cellulose, chitin powder, and sucrose as the single carbon sources. By comparing the growth of the colonies, their abilities to utilize the sugars were analyzed. The diameters of all plates were measured when the overexpressed colony reached the maximum range. The results showed that the WT strains grew normally on every polysaccharide, whereas the overexpressed strains utilized the sugars better than the WT. The growth of overexpressed and WT strains showed obvious differences. The interfered strains could grow normally only on glucose medium. The utilization conditions of all other sugars were much less efficient. Although the conditions of the interfered strain were extremely poor when utilizing the polysaccharides, and the efficiency of the overexpressed strain in utilizing chitin was definitely increased, the results revealed that the difference in efficiency between the overexpressed and WT strains was not as large as in other groups (Figure 4).

These results indicate that *T. asperellum* T4 requires the presence of the ABC-B transporter to utilize different polysaccharides, such as maltose, cellulose, chitin, and sucrose. The overexpressed ABC-B obviously increased the ability of the ABC system to transport polysaccharides. Furthermore, the lack of ABC-B significantly affected its ability to uptake disaccharides. This result matches the growth conditions of *Streptomycetes* found in a previous study [25]. In that study, the *msik* gene, which encodes part of the ABC transporter in *Streptomycetes*, was mutated, and the ability of the bacteria to utilize polysaccharides was also severely damaged. This is because the ABC-B protein is responsible for transporting polysaccharides into the cell. Above all, this result suggests that, in eukaryons, the exporter of the ABC transporter also plays the role of an importer as a supplementary function.

### 2.4. ABC-B Is Essential for (GlcNAc)_2_ Transportation

**Figure 5:**
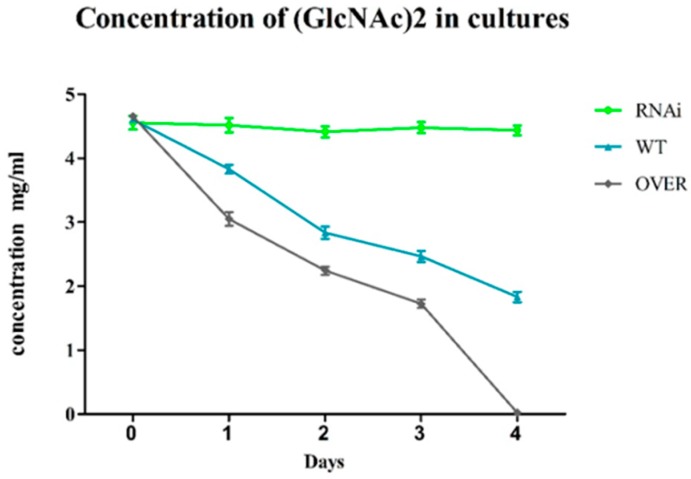
High performance liquid chromatography detection of the (GlcNAc)_2_ concentration. The x-axis represents the number of days of incubation and the y-axis represents the (GlcNAc)_2_ concentration. The samples were analyzed using HPLC every 24 h.

To detect the uptake ability of (GlcNAc)_2_ after interference and overexpression, 99% pure (GlcNAc)_2_ was used as the only carbon source in the medium. The same original (GlcNAc)_2_ concentration was added to the three groups: the ABC-B-interfered strain, WT, and the ABC-B overexpressed strain. Samples were taken from culture supernatants every 24 h after four days of incubation. The (GlcNAc)_2_ concentration of each sample was determined using HPLC. Samples with patterns of decreasing (GlcNAc)_2_ concentrations were compared with those of other samples. The results revealed that the interfered group showed little (GlcNAc)_2_ degradation because only 22% of the ABC-B protein remained, and due to the stability of the chitobiose Na salt form. Concentrations of overexpressed and interfered strains were lower on days 2 and 3. On day 4, no (GlcNAc)_2_ was detected using HPLC in the culture of overexpressed strain, whereas 30% remained in the WT group. The (GlcNAc)_2_ transportation efficiency of the ABC overexpressed strain was evidently improved compared to WT. The (GlcNAc)_2_ concentration in the RNAi group hardly changed, indicating that a lack of the ABC-B transporter markedly affected its uptake ability of (GlcNAc)_2_ (Figure 5). This demonstrates that the overexpressed ABC-B protein improved the fungus’s ability to uptake (GlcNAc)_2_. Thus, the ABC-B transporter plays an important role as an importer in (GlcNAc)_2_ transportation. Intracellular fluids from the three groups were also extracted and analyzed using HPLC. However, no (GlcNAc)_2_ was detected. Scarcelli studied GlcNAc using [C^14^]GlcNAc and a scintillation counter and concluded that *Saccharomyces cerevisiae* transports GlcNAc at a rate of 0.016–0.445 pmol per min [32].

The ability of the ABC transporters to transport GlcNAc and (GlcNAc)_2_ in *Streptococcus pneumoniae* and *Streptomyces coelicolor* has been studied previously [25,33]. However, whether ABC transporters in other microorganisms bear the same function remains unknown. The improved uptake efficiency of (GlcNAc)_2_ and the growth conditions of polysaccharides suggest that ABC-B plays roles as both an importer and an exporter in *T. asperellum* T4.

### 2.5. The ABC-B Transporter Is Essential for Chitinase Induction

**Figure 6:**
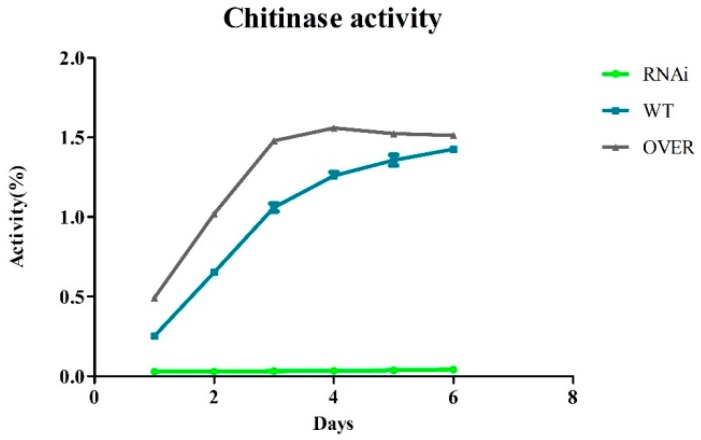
Enzyme activity of chitinase from the extracellular fluid of *T. asperellum* T4.

Several studies have reported that chitobiose and chitinase have a mutual induction phenomenon. Chitinase plays a crucial part in chitobiose production [34]. To determine whether chitobiose plays the same role as the chitinase inducer in T4, the activity of ECH42 was analyzed. ECH42 is an important agent in chitin degradation and biocontrol. It can only be induced by an insoluble chitin, such as (GlcNAc)_2_ or colloidal chitin. Therefore, using it as a model enzyme can reduce the inductive effect of chitin powder. The reduced chitin utilization efficiency of the interfered strain suggests that ABC-B is a key protein for the transportation of chitin or chitosan through the membrane into the cell. Similar to the wild-type, both interfered and overexpressed strains were incubated in MM supplemented with colloidal chitin as the only carbon source. The activity of chitinase in the three groups was detected after 6 days of incubation using colloidal chitin as the substrate. The (GlcNAc)_2_ concentration in the culture was detected using HPLC, and this indicated that the lack of ABC-B transporter had an obvious effect on the uptake of (GlcNAc)_2_. The interfered strains almost completely lost the ability to transport chitobiose (Figure 5). The crude enzyme activity curve of chitinase indicated that the enzyme production of the overexpressed strain is more efficient than that of the WT (Figure 6), and the enzyme activity of the ABC-B-interfered strain remained negligible after day 1 of incubation, which matched the results of the colony culture.

**Figure 7:**
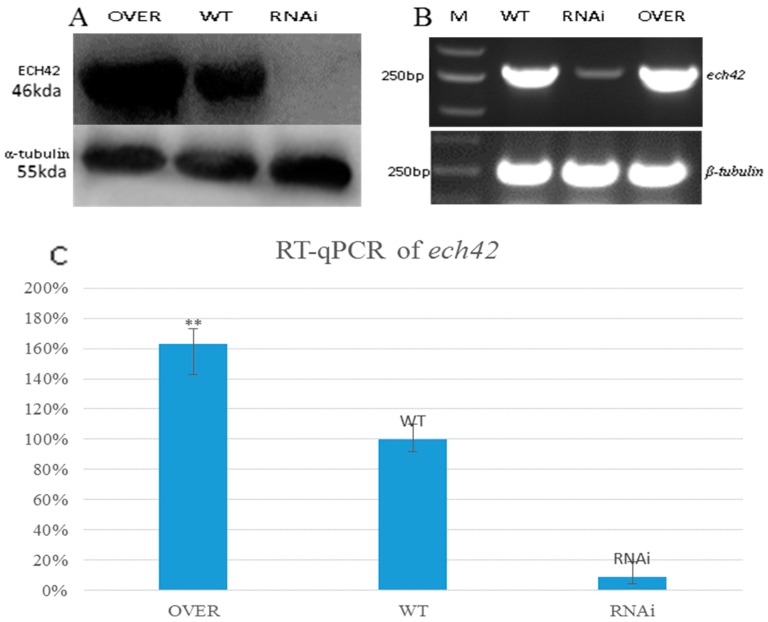
Western blot results for ECH42. Semi-quantitative and Realtime-qPCR results for ECH42 with (GlcNAc)_2_ as the only carbon source and the reference (α-tubulin). (**A**) Western blot result for ECH42 and α-tubulin. OVER: Western blot result for ECH42 from the ABC-B-overexpressed strain, OVER-1. WT: ECH42 protein level from WT-2. RNAi: Western blot result for the interfered strain, RNAi-1. (**B**) Semi-PCR results for ECH42 and β-tubulin. M: 2000 bp DNA marker. WT: ECH42 semi-PCR result for WT-2. RNAi: ECH42 from the ABC-B-interfered strain, RNAi-1. OVER: ECH42 from the ABC-B-overexpressed strain, OVER-1. (**C**) Histogram of the ABC-B RT-qPCR (*p* < 0.01).

Differences in the activity of the interfered and overexpressed ABC-B strains further suggested that (GlcNAc)_2_ is essential for chitinase induction in *T. asperellum* T4. The expression level of ECH42 in the overexpressed strain, interfered strain, and WT when (GlcNAc)_2_ was the only carbon source was compared. Under (GlcNAc)_2_ conditions, the expression level of ECH42 was 160% and 9% that of WT in the overexpressed and interfered strains, respectively (Figure 7C). On a genetic level, ECH42 was barely detected in the interfered strain (Figure 7B). The Western blot result of ECH42 showed that the expression level of ABC-B was markedly improved in the overexpressed strain. Based on the Image J results, since the ABC-B of the interfered strain could not be detected, the expression level of the overexpressed strain was determined to be 175% that of the WT. This was in accordance with the qPCR result (Figure 7A,B) and growth conditions (Figure 4).

Although the ability of ABC-B overexpressed strains to utilize polysaccharides (Figure 4) did not show the three-fold increase demonstrated by the western blot results of overexpressed strains (Figure 3B), the ability to uptake (GlcNAc)_2_ and produce ECH42 with (GlcNAc)_2_ as a single carbon source was dramatically increased. The increases in the activity of crude chitinase and the expression level of ABC-B in the overexpressed strain did not increase as promisingly, as shown in Figure 4. This may indicate that the uptake system of the ABC transporter has an inhibitory mechanism or, more likely, because of its main function of exporting hazardous substances, rather than transporting substrates into cell, the (GlcNAc)_2_ transportation of the ABC-B is limited. The WB result for ECH42 further revealed that the (GlcNAc)_2_ concentration is related to ECH42 expression, which indicates that (GlcNAc)_2_ is an inducer of ECH42 in *T. asperellum* T4. The ABC-B protein is the most essential importer for *T. asperellum* in the uptake of chitobiose. However, whether chitobiose directly binds to the ECH42 promoter region or stimulates the expression of ECH42 through another pathway requires further research.

## 3. Materials and Methods

### 3.1. Bacteria, Fungus, Plasmids, and Culture Conditions

*T. asperellum* T4 was isolated from soil samples from Mishan City, China (preservation number CGMCC3.14975). *Escherichia coli* Top10 was used as the competent cell. T4 was cultured in a Minimal Medium (MM culture comprised of 1.0 g NH_4_NO_3_, 0.5 g KH_2_PO_4_, 1.5 g Na_2_HPO_4_, 1.0 g NaCl, and 0.2 g MgSO_4_·7H_2_O per liter, with a pH of 7.2) with glucose, (GlcNAc)_2_, or other polysaccharides as the single carbon source. The *E. coli* strains Top 10, pBargpe, and pSilent carrier were cultured in LB medium (10 g NaCl, 10 g tryptone, and 5 g yeast extraction per liter, with a pH of 7.0) with antibiotics. T4 was incubated at 28 °C, and bacteria were incubated at 37 °C in a rotating shaker at 180 rpm. Plasmids (pBargpe-Hygro-1 and Psilent-1) were purchased from Miaoling Company, Wuhan city, China.

### 3.2. Extraction of RNA, PCR Amplification, and RT-qPCR

The mycelia cell wall of T4 was embrittled, dehydrated with liquid nitrogen, and triturated into powder after four days of incubation in MM culture. Mycelia powder was processed with Trizol reagent, separated by chloroform, and precipitated by isopropanol. The sediment was washed with absolute ethyl alcohol and dissolved in RNase-free water. Later, it was treated with RNase-free DNase I (Thermo Fisher™, Shanghai, China) to remove any remnant genomic DNA fragments. Then, total RNA was conversed into cDNA with Toyobio™ (Shanghai, China) (1 μg RNA). The RNA quality was analyzed using agarose gel electrophoresis, and a five-fold diluted cDNA was used as the template for RT-qPCR. For RT-qPCR, Go Taq qPCR Master Mix (Promega™, Beijing, China) and the β-tubulin gene (GenBank: Accession KF595249.2) of *T. asperellum* were used as internal controls. The relative abundance of *ABC* and *ECH42* cDNA was standardized using the expression levels of the β-tubulin gene. PCR was conducted using the *Pfu* DNA polymerase Ex taq. The regular amplification process of the *ABC* sequence was as follows: 2 min at 95 °C, 30 cycles of 1 min at 95 °C, 2 min at 60 °C, 2 min at 72 °C, 10 min at 72 °C, and then a hold at 4°C. The annealing temperature for *ECH42* was 57 °C. The number of cycles was reduced to 24 for both sequences for semi-quantitative PCR. The RT-qPCR steps were as follows: 95 °C for 1 min, the following two steps were then repeated for 40 cycles: (1) 95 °C for 14 s and (2) 60 °C for 1 min, 95 °C for 15 s, 60 °C for 1 min and 95 °C for 15 s. All RT-qPCR and Western blot results were quantified with software.

### 3.3. Plasmid Construction

#### 3.3.1. Construction of the Interference Plasmid

**Figure 8:**
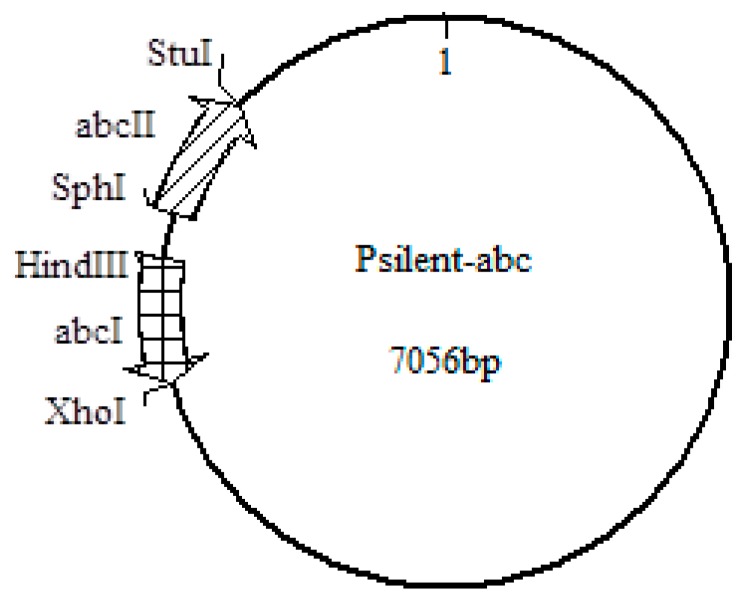
Construction of the Psilent-*ABC* plasmid. All four enzyme sites are from the plasmid. ABCI and ABCII are parts of the hairpin structure.

The amino acid sequence of the ABC transporter of *T. asperellum* (GenBank: JN808453.1) was analyzed using the online Simple Modular Architecture Research Tool (SMART) (available online: http://smart.embl-heidelberg.de/) to confirm the essential functional areas of the ABC-B protein that had been interfered. The primers were designed based on the sequence selected, and the target sequence was 500 bp long, which covered one main area of the transmembrane region and one ATP-binding domain. The enzyme sites were *HindIII* and *XhoI* for ABCI and *SphI* and *StuI* for ABCII on the pSilent plasmid as Figure 8 shown. The primers designed for *T. asperellum* T4 in this paper are shown in Table 1.

#### 3.3.2. Construction of the Overexpression Plasmid

**Figure 9:**
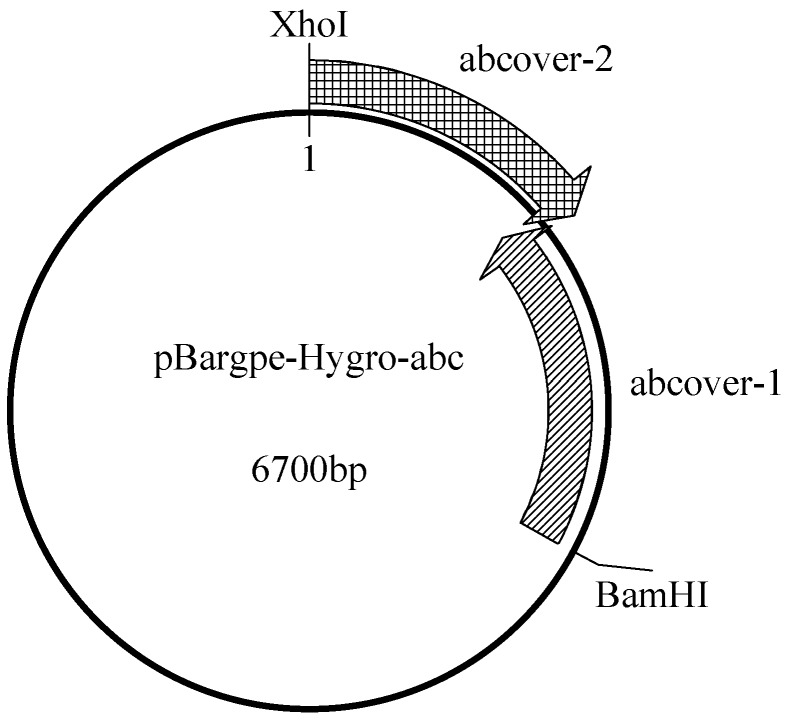
Construction of the pBargpe-Hygro-*ABC* plasmid. Both enzyme sites are from the plasmid. ABCOVER-1 and ABCOVER-2 have a 25 bp overlap at their junction.

Two pairs of primers were designed based on the mRNA sequence of the *T. asperellum* ABC-B transporter family protein base to reduce the mutation probability in one-step PCR. The two sequence parts had a 25 bp mutual zone to allow seamless assembly. These two sequences were connected as per the instructions provided on the ClonExpress II One Step Cloning Kit. Judging by the direction of pBargpe expression, the enzyme sites at the 5′ upstream position and the 3′ downstream position were *BamHI* and *XhoI* as Figure 9 shown.

Both the interference and overexpression plasmids and their respective target sequences were linked using T4 ligase and the competent cell of *E. coli*, Top10, for transformation. Transformants were selected using ampicillin^+^ LB agar plates, and plasmids were confirmed through double enzyme digestion before being forwarded for sequencing.

### 3.4. Construction of Protoplasts and Transformants

After constructing the plasmids pSilent-*ABC* and pBargpe-Hygro-*ABC*, promega mediated plastid transformation was performed. Protoplasts were collected according to the steps described by Anthony [35], with the following modifications. After three days of incubation, mycelia were digested using lyticase (Guangdong Institute of Microbiology) in an orbital shaker incubator at 30 °C and 120 rpm. After filtration with double-level lens paper, the supernatant was centrifuged at 4000 rpm and 4 °C for 5 min. After dumping the supernatant, sediments were resuspended in 0.06 mol/L STC (27.3 g sorbitol, 0.75 g Tris-HCl, 0.13 g CaCl_2_, and 125 mL water, with a pH of 7.5). This step was repeated, and the concentration of protoplasts was calculated after another resuspension in 200 μL STC. After this, 10 μg of pSilent-*ABC* or pBargpe-Hygro-*ABC* was added into the STC and incubated for 20 min at 0 °C. After adding 50% PEG4000 (50 g PEG4000, 8.48 g Tris-HCl, 0.11 g CaCl_2_, and 100 mL water, with a pH of 7.5) into the protoplast–plasmid mixture, the new mixture was incubated at 0 °C for 20 min. This mixture was added to 10 mL of regeneration medium (0.1 g yeast extract and peptone, 3.42 g sucrose, 0.15 g agar, and 10 mL water, with a pH of 7.0) as the first layer and covered with a second layer of selection medium (0.1 g agar, 10 mL PDA, and 30 μg hygromycin B) at 28 °C. This was incubated for 12 h as an inverted plate. The growth situation of transformants was observed after addition on the second layer for 24 h, and a single colony was isolated.

### 3.5. Detection of the (GlcNAc)_2_ Concentration

High-performance liquid chromatography (HPLC) was applied to detect the changes in the transportation efficiency of (GlcNAc)_2_ after genomic engineering. Given the chemical properties of (GlcNAc)_2_, a Shimadzu LC-20AD, Shodex NH2P-50 4E 4.6 × 250 mm, 5 µm NH_2_ column was used at 30 °C with a flow rate of 1.0 mL/min and a mobile phase of 75% acetonitrile and 25% water. The (GlcNAc)_2_ concentration was calculated using the corresponding peak areas based on the standard curve of (GlcNAc)_2_.

### 3.6. Western Blot

To detect the changes in the protein expression levels of ABC-B and ECH42 in the overexpressed, wild-type (WT), and interfered strains, rabbit anti-ABC-B1 and anti-ECH42 antibodies (General Biosystems Co, Ltd., Chuzhou, China) were chosen as primary antibodies. Goat anti-rabbit antibodies were chosen as secondary antibodies, and the α-tubulin antibody #2144 (Cell Signalling Technology™, Shanghai, China) was used as a reference. All results were analyzed using Image J software (National Institutes of Health, Bethesda, MD, USA).

### 3.7. Extraction of Fungi Wall Protein

After four days of incubation, the mycelia were centrifuged at 4000 rpm for 10 min. This step was repeated until the supernatant became clear. Cell wall sediment was ground chemically (2% CTAB and 2-mercaptoethanol) and ultrasonically until the wall-broken rate was 95%. Solutions of 5%, 2%, and 0.9% NaCl were added into the sediment successively and centrifuged at 4000 rpm for 10 min. Each step was repeated three times. The sediment was washed and resuspended in water and then dried in a lyophilizer.

### 3.8. Chitinase Activity

(GlcNAc)_2_ disturbs the DNS reaction because of its reducing sugar nature, so the chitinase in the (GlcNAc)_2_ environment must be separated before detecting its activity. Therefore, saturated ammonium sulphate was added to the extracellular fluid of the culture before centrifugation for 30 min at 8000 rpm. The sediment was dissolved in water and dialyzed through a dialysis bag in water. The mixture was centrifuged, and the sediment was washed with 2 mL of acetone. This step was repeated several times, and the sediment was dissolved in Tris-EDTA (TE) buffer solution in a proportion of 100 to 1 (100 mL extracellular fluid to 1 mL TE). One unit of chitinase activity was defined as the amount of enzyme that degraded 1 μmol of colloidal chitin in 1 min at 37 °C.

## Figures and Tables

**Table 1:** Primers used in this study.

Category	Name	Content	Product Size (bp)	Function
Overexpression primer	TAABCP1-5	CGGATCCATGGCAGACCACGGTGAAGAGAAAG	2451 ABCOVER-1	*ABC* upstream sequence
	TAABCP1-3	GGCATGCCTTCTGCCCTCAGTATGC		
	TAABCP2-5	CCTCGAGAGCCCTTGTGGTCTCG	1923 ABCOVER-2	*ABC* downstream sequence
	TAABCP2-3	AAGGCCTAGCCCTTGTGGTCTCG		
Interference primer	TAABCI-5	CAAGCTTCTTCTGCCCTCAGTATGC	500 ABCI	Crucial section of ABC to interfere
	TAABCI-3	CCTCGAGAGCCCTTGTGGTCTCG		
	TAABCII-5	GGCATGCCTTCTGCCCTCAGTATGC	500 ABCII	
	TAABCII-3	AAGGCCTAGCCCTTGTGGTCTCG		
*ABC* qPCR primer	TAABCq-5	CTCAGACCCACTCTCCGAA	280	Expression level of *ABC*
	TAABCq-3	GGGTCTGAGAGCGGAAAA		
*ECH42* qPCR primer	Ech42q-5	CAACTGGGGTATCTATGGC	250	Expression level of *ECH42*
	Ech42q-3	TTGCTGCAGAAGGGAAG		
β-tubulin qPCR primer	Tubulin-5	CACCTTCCTCCATACCCTCACC	200	Reference gene
	Tubulin-3	TGTCCAACACCACCGCCATC		

## References

[1] Ruocco M., Lanzuise S., Vinale F., Marra R., Turrà D., Woo S.L., Lorito M. (2009). Identification of a New Biocontrol Gene in *Trichoderma atroviride*: The Role of an ABC Transporter Membrane Pump in the Interaction with Different Plant-Pathogenic Fungi. Mol. Plant-Microbe Interact..

[2] Kim J., Wu S., Tomasiak T.M., Mergel C., Winter M.B., Stiller S.B., Robles-Colmanares Y., Stroud R.M., Tampe R., Craik C.S. (2015). Subnanometre-resolution electron cryomicroscopy structure of a heterodimeric ABC exporter. Nature.

[3] Shepherd M. (2015). The CydDC ABC transporter of Escherichia coli: New roles for a reductant efflux pump. Biochem. Soc. Trans..

[4] Ponte-Sucre A. (2007). Availability and applications of ATP-binding cassette (ABC) transporter blockers. Appl. Microbiol. Biotechnol..

[5] Dean M., Hamon Y., Chimini G. (2001). The human ATP-binding cassette (ABC) transporter superfamily. J. Lipid Res..

[6] Dean M., Annilo T. (2005). Evolution of the ATP-binding cassette (ABC) transporter superfamily in vertebrates. Ann. Rev. Genom. Hum. Gen..

[7] Ter Beek J., Guskov A., Slotboom D.J. (2014). Structural diversity of ABC transporters. J. Cell Biol..

[8] Wang T., Fu G., Pan X., Wu J., Gong X., Wang J., Shi Y. (2013). Structure of a bacterial energy-coupling factor transporter. Nature.

[9] Rees D.C., Johnson E., Lewinson O. (2009). ABC transporters: The power to change. Nat. Rev. Mol. Cell Biol..

[10] Rice A.J., Park A., Pinkett H.W. (2014). Diversity in ABC transporters: Type I, II and III importers. Crit. Rev. Biochem. Mol. Biol..

[11] Locher K.P. (2009). Structure and mechanism of ATP-binding cassette transporters. Philos. Trans. R. Soc. B Biol. Sci..

[12] Kang J., Park J., Choi H., Burla B., Kretzschmar T., Lee Y., Martinoia E. (2011). Plant ABC transporters. Arab. Book/Am. Soc. Plant Biol..

[13] Borghi L., Kang J., Ko D., Lee Y., Martinoia E. (2015). The role of ABCG-type ABC transporters in phytohormone transport. Biochem. Soc. Trans..

[14] Lee M., Choi Y., Burla B., Kim Y.Y., Jeon B., Maeshima M., Yoo J.Y., Martinoia E., Lee Y. (2008). The ABC transporter AtABCB14 is a malate importer and modulates stomatal response to CO_2_. Nat. Cell Biol..

[15] De Marcos Lousa C., Van Roermund C.W., Postis V.L., Dietrich D., Kerr I.D., Wanders R.J., Baldwin S.A., Baker A., Theodoulou F.L. (2013). Intrinsic acyl-CoA thioesterase activity of a peroxisomal ABC transporter is required for transport and metabolism of fatty acids. Proc. Natl. Acad. Sci. USA.

[16] Howell C.R. (2003). Mechanisms employed by *Trichoderma* species in the biological control of plant diseases: The history and evolution of current concepts. Plant Dis..

[17] Dong X., Zhao Y., Ran X., Guo L., Zhao D.G. (2017). Overexpression of a New Chitinase Gene EuCHIT2 Enhances Resistance to *Erysiphe cichoracearum* DC in Tobacco Plants. Int. J. Mol. Sci..

[18] Zheng Y., Wang X., Liu S., Zhang K., Cai Z., Chen X., Zhang Y., Liu J., Wang A. (2018). The Endochitinase of Clonostachysrosea Expression in *Bacillus amyloliquefaciens* Enhances the *Botrytis cinerea* Resistance of Tomato. Int. J. Mol. Sci..

[19] El-Katatny M.H., Gudelj M., Robra K.H., Elnaghy M.A., Gübitz G.M. (2001). Characterization of a chitinase and an endo-ß-1,3-glucanase from *Trichoderma harzianum* Rifai T24 involved in control of the phytopathogen *Sclerotium rolfsii*. Appl. Microbiol. Biotechnol..

[20] Langner T., Göhre V. (2016). Fungal chitinases: Function, regulation, and potential roles in plant/pathogen interactions. Curr. Genet..

[21] Jung W.J., Park R.D. (2014). Bioproduction of chitooligosaccharides: Present and perspectives. Mar. Drugs.

[22] Yang S., Fu X., Yang Q., Guo Y., Liu Z., Jiang Z. (2016). Cloning, expression, purification and application of a novel chitinase from a thermophilic marine bacterium *Paenibacillus barengoltzii*. Food Chem..

[23] Kzhyshkowska J., Yin S., Liu T., Riabov V., Mitrofanova I. (2016). Role of chitinase-like proteins in cancer. Biol. Chem..

[24] Da Silva A.F., García-Fraga B., López-Seijas J., Sieiro C. (2017). Optimizing the expression of a Heterologous chitinase: A study of different promoters. Bioengineered.

[25] Saito A., Fujii T., Shinya T., Shibuya N., Ando A., Miyashita K. (2008). The msiK gene, encoding the ATP-hydrolysing component of *N*,*N*′-diacetylchitobiose ABC transporters, is essential for induction of chitinase production in *Streptomyces coelicolor* A3(2). Microbiology.

[26] Niu X., Liu C.C., Xiong Y.J., Yang M.M., Ma F., Liu Z.H., Yuan S. (2016). The Modes of Action of ChiIII, a Chitinase from Mushroom *Coprinopsis cinerea*, Shift with Changes in the Length of GlcNAc Oligomers. J. Agric. Food Chem..

[27] Brunner K., Montero M., Mach R.L., Peterbauer C.K., Kubicek C.P. (2003). Expression of the ech42 (endochitinase) gene of *Trichoderma atroviride* under carbon starvation is antagonized via a BrlA-like cis-acting element. FEMS Microbiol. Lett..

[28] Theodoulou F.L., Kerr I.D. (2015). ABC transporter research: Going strong 40 years on. Biochem. Soc. Trans..

[29] Walker J.E., Saraste M., Runswick M.J., Gay N.J. (1982). Distantly related sequences in the α- and β-subunits of ATP synthase, myosin, kinases and other ATP-requiring enzymes and a common nucleotide binding fold. EMBO J..

[30] Yazaki K. (2006). ABC transporters involved in the transport of plant secondary metabolites. FEBS Lett..

[31] Verrier P.J., Bird D., Burla B., Dassa E., Forestier C., Geisler M., Klein M., Kolukisaoglu U., Lee Y., Martinoia E. (2008). Plant ABC proteins: A unified nomenclature and updated inventory. Trends Plant Sci..

[32] Scarcelli J.J., Colussi P.A., Fabre A.L., Boles E., Orlean P., Taron C.H. (2012). Uptake of radiolabeled GlcNAc into Saccharomyces cerevisiae via native hexose transporters and its in vivo incorporation into GPI precursors in cells expressing heterologous GlcNAc kinase. FEMS Yeast Res..

[33] Robb M., Hobbs J.K., Woodiga S.A., Shapiro-Ward S., Suits M.D., McGregor N., Brumer H., Yesilkaya H., King S.J., Boraston A.B. (2017). Molecular Characterization of N-glycan Degradation and Transport in Streptococcus pneumoniae and Its Contribution to Virulence. PLoS Pathog..

[34] Le B., Yang S.H. (2018). Characterization of a chitinase from *Salinivibrio* sp. BAO-1801 as an antifungal activity and a biocatalyst for producing chitobiose. J. Basic Microbiol..

[35] Anthony P., Davey M.R., Power J.B., Washington C., Lowe K.C. (1994). Synergistic enhancement of protoplast growth by oxygenated perfluorocarbon and Pluronic F-68. Plant Cell Rep..

